# Extensive Amyloid Purpura: An Unusual Presentation of Myeloma-associated Light Chain Amyloidosis

**DOI:** 10.2340/actadv.v103.13367

**Published:** 2023-06-27

**Authors:** Nor Fazil JABER, Ulrika THELANDER, Philip CURMAN

**Affiliations:** 1 Dermato-Venereology Clinic, Karolinska University Hospital, Eugeniavägen 3, SE-17164 Stockholm; 2 Department of Immunology, Genetics and Pathology, Uppsala University, Uppsala; 3 Dermatology and Venereology Division, Department of Medicine (Solna); 4 Department of Medical Epidemiology and Biostatistics, Karolinska Institutet, Stockholm, Sweden

Systemic amyloidosis is a rare disorder characterized by atypical protein deposits in various tissues and organs (1). The main forms are light chain (AL) amyloidosis and transthyretin-related amyloidosis (2), but, to date, 18 different proteins have been shown to cause systemic amyloidosis in humans (3). AL amyloidosis may be idiopathic or associated with myeloma, with several cutaneous manifestations reported (4). We report here a rare case of extensive purpura as a cutaneous manifestation of amyloidosis, subsequently identified as myeloma-associated AL amyloidosis.

## CASE REPORT

**Fig. 1:**
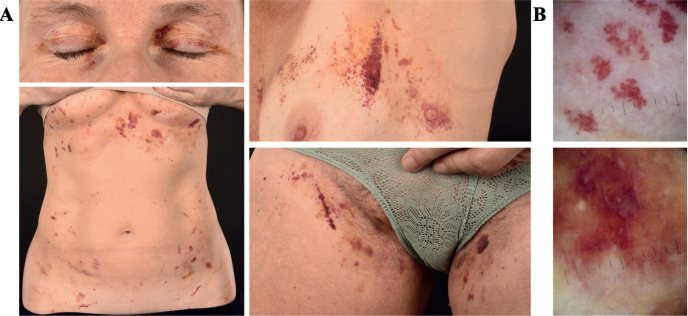
**Clinical picture and dermatoscopy.** (A) Superficial purpura in the periorbital region, anterior trunk, and groin. (B) Dermatoscopy showing characteristic well-defined, non-blanching, red and purple structureless macules and patches. B: Location: left front torso. Magnification: 10x.

**Fig. 2:**
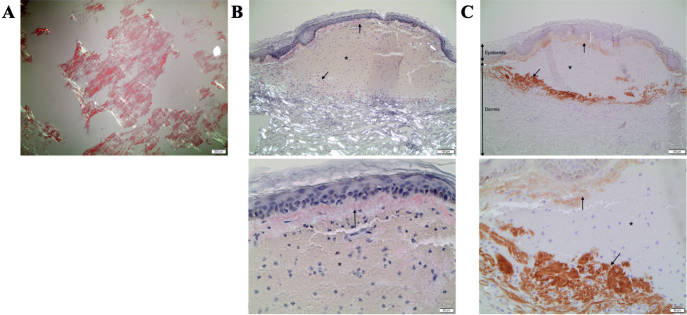
**Histopathology.** (A) Congo staining of abdominal subcutaneous fat showing abundant light chain (AL) amyloid deposits. (B) Skin biopsy Congo staining showing amyloid deposits (*arrows*). (C) Skin biopsy immunohistochemical staining for AL lambda (*arrows*). *Intradermal bleeding. A: 4x. B and C: 10x (upper), 40x (lower).

A 56-year-old woman with a 28-year history of rheumatoid arthritis presented to our clinic with a 12-month history of spontaneous periorbital bleedings and superficial haematomas on the chest and groin. The lesions had subsequently spread to involve the whole anterior trunk and groin (**Fig. 1**A). Examination revealed disseminated macular purpuric lesions in the skin, which was fragile and exhibited bleeding at the slightest trauma. Dermatoscopy showed characteristic signs of purpura (Fig. 1B). The patient also reported generalized weakness, shortness of breath, and intermittent tongue swelling causing difficulties when speaking. A 4-mm punch biopsy displayed extensive bleeding and Congo staining for amyloid depositions was initially considered negative. Blood analyses revealed no significant abnormalities. A clinical suspicion of amyloid purpura led to a deep 8-mm punch biopsy of abdominal subcutaneous fat, which showed abundant AL amyloid deposits (**Fig. 2**A). Re-examination of the skin biopsy stained positive for amyloid with Congo staining as well as with immunohistochemical staining for AL lambda, and intradermal bleeding was seen (Fig. 2B, C). Over a year after the onset of atypical skin symptoms, a myeloma diagnosis was made based on bone marrow aspirate smear. The final diagnosis was myeloma-associated systemic AL-amyloidosis with a rare cutaneous presentation of amyloid purpura. The patient is undergoing treatment with bortezomib and daratumumab, with improving symptoms.

## DISCUSSION

Amyloid purpura is a rare cutaneous presentation of AL amyloidosis, which can be secondary to myeloma (5). Symptoms of amyloidosis are diverse, depending on affected organs. Skin and mucosa involvement occur as primary cutaneous amyloidosis or secondary to systemic amyloidosis. Common cutaneous manifestations in 25% of AL amyloidosis patients include purpura, waxy papules, and ecchymoses. Periorbital haemorrhage (Raccoon-eye sign) is also characteristic of this rare, life-threatening disorder (4). Sometimes, skin lesions may be the only presentation before later-stage organ involvement. A thorough clinical evaluation of the skin with recognition of common and uncommon cutaneous findings of amyloidosis is important for early detection of a possible underlying myeloma, as early initiation of therapy improves the prognosis and survival rate (2).

The clinical variability of systemic amyloidosis, in combination with the relative rarity, makes diagnosis difficult, which may lead to a significant delay in diagnosing a potentially life-threatening underlying myeloma (2). Since identification of amyloid deposits in routine skin biopsy samples may be difficult, as displayed in the current case, subcutaneous abdominal fat biopsy is the preferred method for detecting systemic amyloidosis, exhibiting good diagnostic accuracy in patients with suspected amyloidosis (6). Furthermore, amyloid subtyping through immunohistochemical staining of biopsy samples is important, since it has implications for disease pathogenesis and the choice of therapy (2). In cases with inconclusive initial diagnostic tests, a multidisciplinary approach involving dermatologists, haematologists, and pathologists is recommended to ensure accurate diagnosis and optimal management.

In conclusion, this case underscores the importance of recognizing extensive purpura upon insignificant trauma as a potential indicator of amyloid purpura, which may be secondary to severe myeloma (5). Early recognition, thorough diagnostic evaluation, and timely initiation of treatment are crucial in ensuring optimal patient outcomes and improving the management of this rare and potentially life-threatening condition.
